# Divergent Processing of Cell Stress Signals as the Basis of Cancer Progression: Licensing NFκB on Chromatin

**DOI:** 10.3390/ijms25168621

**Published:** 2024-08-07

**Authors:** Spiros A. Vlahopoulos

**Affiliations:** 1st Pediatric Clinic, University of Athens, 11527 Athens, Greece; sblachop@med.uoa.gr

**Keywords:** chromatin, histone, cell stress, unfolded protein response, inflammation, nuclear factor kappa B, cytokines, chemokines, super enhancer, BET inhibitor

## Abstract

Inflammation is activated by diverse triggers that induce the expression of cytokines and adhesion molecules, which permit a succession of molecules and cells to deliver stimuli and functions that help the immune system clear the primary cause of tissue damage, whether this is an infection, a tumor, or a trauma. During inflammation, short-term changes in the expression and secretion of strong mediators of inflammation occur, while long-term changes occur to specific groups of cells. Long-term changes include cellular transdifferentiation for some types of cells that need to regenerate damaged tissue, as well as death for specific immune cells that can be detrimental to tissue integrity if they remain active beyond the boundaries of essential function. The transcriptional regulator NFκB enables some of the fundamental gene expression changes during inflammation, as well as during tissue development. During recurrence of malignant disease, cell stress-induced alterations enable the growth of cancer cell clones that are substantially resistant to therapeutic intervention and to the immune system. A number of those alterations occur due to significant defects in feedback signal cascades that control the activity of NFκB. Specifically, cell stress contributes to feedback defects as it overrides modules that otherwise control inflammation to protect host tissue. NFκB is involved in both the suppression and promotion of cancer, and the key distinctive feature that determines its net effect remains unclear. This paper aims to provide a clear answer to at least one aspect of this question, namely the mechanism that enables a divergent response of cancer cells to critical inflammatory stimuli and to cell stress in general.

## 1. Introduction

After the function of a tissue is disrupted, inflammation is activated by diverse molecular triggers, which induce the expression of cascades of cytokines and adhesion molecules, permitting a succession of essential molecules and cells to deliver stimuli and functions that mobilize the immune system to clear the cause of the disruption of tissue function, whether this is an infection, a tumor, or a trauma. During inflammation, both short-term changes as well as long-term changes occur, involving specific groups of cells; short-term changes include the expression and secretion of potent mediators of inflammation, which have drastic and mostly local effects, while long-term changes include cellular transdifferentiation for some types of cells that need to regenerate damaged tissue [1,2]. Long-term changes also include death for cells that engage with and destroy the primary cause of inflammation, such as those types of immune cells that can be detrimental to tissue integrity if they remain active beyond the limits of the tissue area that was initially functionally compromised. Especially short-term changes in gene expression during inflammation are terminated by specific negative feedback signals [3]. These negative feedback signals protect local tissue from destruction by excessive inflammation [4].

During cancer, mediators of inflammation often enter a regulatory mode that allows their expression in an almost constitutive manner, which is typically accomplished by changes in the chromatin that may extend several thousand kilobase pairs away from their transcription start site; such regulatory elements are enhancers that are characterized by increased histone H3 acetylation at the N-terminus on the lysine residue at position 27 [5]. This transcriptional activity is associated with the bromodomain-containing factor BRD4, which also binds to acetylated lysine-310 of the subunit RelA (p65) of the activity of transcription factor NFκB, maintaining persistently active NFκB in malignant tumors [6]. The transcriptional regulator NFκB is a key factor that enables some of the fundamental changes in gene expression during inflammation, as well as during tissue development, both in physiology as well as in pathology.

During the recurrence of malignant disease, diverse alterations enable the growth of cancer cell clones that are substantially resistant to therapeutic intervention and to the immune system [7,8,9]. Cellular stress and chromatin remodeling have been linked to a certain degree of “lineage infidelity” that is characteristic of recurrent cancer [10,11,12]. A number of those chromatin alterations occur due to significant defects in feedback signal cascades that control the activity of NFκB [13,14,15].

Although the transactivator NFκB is a protein dimer that can be composed of different subunits, which are activated through at least three pathways, canonical, non-canonical, and atypical [16,17,18,19,20], in this paper we focus mainly on the p65/p50 heterodimer that is activated by the canonical pathway. From the diverse types of posttranslational modifications that activate the p65 (RelA) subunit [21], here, we mainly direct our attention to the phosphorylation on its serine amino acid residue in position 276, because this modification allows p65 to recruit chromatin-modifying protein complexes that allow for activating the expression of genes by increasing chromatin accessibility [22]. In contrast, these genes may be constitutively accessible in cancer “stem-like” cells due to the presence of chromatin modifications in the basal state. This fact alters the way that these malignant cells respond to cell stress, and it has not yet been adequately addressed. Other aspects of NFκB function in cancer, such as the different interactions of NFκB with oncogenes, and the resulting effects on cell fate and cell phenotype have been exhaustively studied and reviewed elsewhere, [23,24,25,26,27,28,29,30,31,32,33] and will only be mentioned here in connection with the key distinguishing feature of relapsed cancer, namely the abnormal response to NFκB inducing cell stress.

## 2. Cell Stress Enables Diverse Events That Can Be Divided into Restricted and Inflammatory

Cell stress can be broadly viewed as a metabolic imbalance that may have a variety of causes and that leads to diverse cascades of events that are either restricted to the cell undergoing the disruption of metabolism, or communicated to other cells and extracellular structures in a manner that helps the organism adjust to a potentially adverse condition [34,35,36,37]. Failure of the stress response can lead to defects in tissue structure, function, and development [38]. The connection between metabolic imbalance and stress is also manifest at the level of a clinical meta-analysis [39].

Cell stress activates two main types of responses: the endogenous response, which aims to absorb the impact of cell stress, and inflammation, which aims to mobilize several different cell types that can have effects on the entire organism. The endogenous response to cell stress involves adaptive triggers of mechanisms for macromolecule degradation, which recycle damaged components or misfolded proteins primarily through the lysosome, enable the cell to reuse amino acids and other building blocks, and ultimately to survive by conserving nutrients and energy and preventing further damage [40,41,42]. This way, by containing the damage response within the cell, the endogenously contained stress is restricted. Restricted cell stress prevents the cause of metabolic imbalance from disrupting tissue function [43].

## 3. Switch from Cell Stress to Inflammation

Conditions that are not resolved in the cell and lead to imbalanced generation of oxidized molecules trigger inflammation, and this may lead to chronic conditions [44]. Inflammation is also directly triggered by molecular patterns that are generated from dead cells or bacterial or viral constituents [45,46,47]. The primary response to inflammatory triggers is the expression of molecules that enable progression of the signal cascades which permit completion of the sequence of events that are needed to resolve the cause of functional disruption in the tissue. However, persistent activation of inflammatory gene expression may lead to defective signal processing in cells and chronic inflammation [44].

While persistent triggers of cell stress cause chronic inflammation, the activation of mechanisms that permit cell survival out of pace with the function of host tissue and in the presence of mutations in key regulators of cell growth sets the stage for cancer [48]. There are multiple mechanisms in place that generate signals to terminate inflammation and to stem cancer growth; both are rooted in the function of the immune system [49]. In particular, the activation of inflammation triggers signal cascades that inevitably lead to production of anti-inflammatory mediators and the growth of abnormal cells triggers signals for destruction by the immune system. Central to both processes is the activation of the transcription factor NFκB, a dimer of proteins of the Rel family that is held inactive in the cytoplasm by proteins of the I kappa B family [50]. Upon phosphorylation by IKK complex proteins and induction of I kappa B proteolysis, the Rel dimer enters the nucleus to activate the transcription of diverse genes; a number of those genes provide feedback to NFκB, which is decisive for the net effect of the activation [51]. Importantly, the I kappa B alpha (IκBα)-encoding gene *NFKBIA* does not have a nucleosome-blocked transcription start site; thus, NFκB activates this negative feedback gene regardless of activating phosphorylation on the Rel subunits, while inflammatory genes and most other downstream targets require, for example, phosphorylation of RelA serine residue 276,which enables the recruitment of histone-modulating complexes [52,53,54]. NFκB signaling is restricted by negative feedback control mechanisms that operate at every level of the NFκB-activating pathways [55]. A number of protein phosphatases restrict NFκB signaling at multiple levels, and normally cell stress inactivates specific phosphatases to permit NFκB signaling [56,57]. For example, superoxide inactivates protein phosphatase 2A by nitrative modification of its substrate-binding subunit B56γ [58]. In acute myeloid leukemia (AML), overexpression of protein phosphatase 2A inactivator SET is associated with a poor prognosis [59].

**Figure 1:**
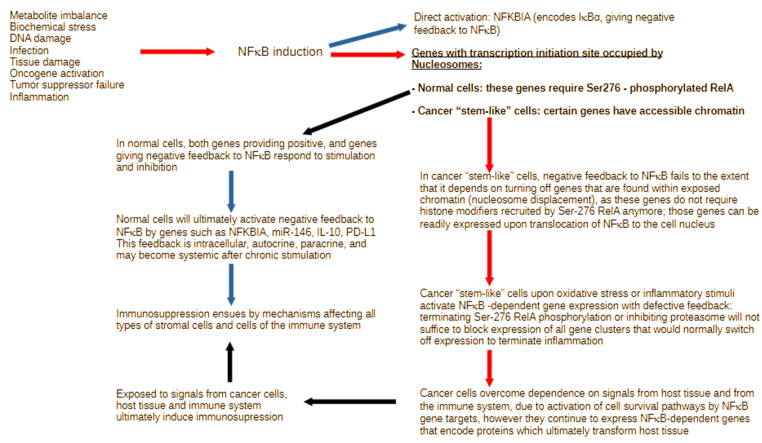
In most cells, a variety of disruptions in tissue or cellular function activate NFκB posttranslational modifications and nuclear translocation, enabling cells to respond by expressing the genes that are needed to resolve the primary cause of stress. The NFKBIA (IκBα) gene is readily activated by NFκB and provides negative feedback. However, most other NFκB-driven genes (underlined bold text) require specific phosphorylation of Rel subunits (bold font), which recruit histone-remodeling complexes to increase chromatin accessibility (black arrows). The latter is not required for certain genes in the exposed chromatin of cancer “stem-like” cells. These cells are, therefore, permitted to express genes detrimental to the host tissue (red arrows). On the one hand, these cancer “stem-like” cells respond differently to cell stress and inflammation, and, on the other hand, their unrestricted expression of key modulators ultimately leads to changes in the host tissue and the immune system. The impact of such changes in gene regulation can have critical effects on tissue function. Blue arrows indicate expression and impact of genes that protect host tissue from excessive inflammation.

Otherwise, in most cells, prolonged, unresolved cell stress inactivates specific negative feedback modulators of NFκB, such as phosphatases, permitting the accumulation of activating posttranslational modifications on the Rel subunits, which trigger the assembly of chromatin-modifying complexes; depending on the duration of cell stress, the result may range from acute to chronic inflammation [60,61,62,63]. However, in cancer “stem-like” cells with exposed chromatin on certain cancer drivers, the complete set of posttranslational modifications on NFκB is not required, meaning that those cancer cells will readily express the cancer driver genes with accessible chromatin upon NFκB induction under favorable metabolic conditions, without the requirement for a complete set of activating modifications on NFκB (Figure 1).

A number of the NFκB-mediated signal cascades that are activated by immunity to suppress tumor growth may actually lead to the opposite effect under the influence of genetic and microenvironmental factors [64]. The main root of cancer cell survival is the divergent signal processing between cancer and stromal cells, especially in respect to NFκB [65]. The divergence in stress signal processing between cancer and stroma is more evident in leukemia and largely concerns responses to inflammatory mediators [66,67]. Crosstalk between the tumor and stroma is largely influenced by endoplasmic reticulum stress, with the unfolded protein response (UPR) as a key trigger [68]. As a condition that modulates tumor development, excessive cellular stress, and endoplasmic reticulum stress in particular, activate multiple mechanisms that end up supporting cancer growth [69]. This cancer-supporting function is due to the inevitable immunosuppression that occurs after excessive cell stress and inflammation [70].

Furthermore, cell stress can be directly caused by chemotherapy or transmitted to the tumor stroma either by inflammatory mediators or extracellular vesicles; the tumor stroma is thereby conditioned to support the survival of cancer cells or aggressive cancer phenotypes. Under the influence of chemotherapy, cancer-associated fibroblasts adopt a “senescence-associated secretory phenotype” that enhances tumor cell growth and invasiveness [71,72]. In general, NFκB has a documented role in the process of adoption of the “senescence-associated secretory phenotype” that entails secretion of inflammatory mediators [73].

In AML, extracellular vesicles practically transmit endoplasmic reticulum stress in vivo from the AML xenograft to the bone marrow stroma, whereby the unfolded protein response drives osteolineage differentiation of mesenchymal stem cells [74]. The transmission of extracellular vesicles helps AML cells to remodel the bone marrow stroma [75].

## 4. Cellular Stress Acts as a Transcriptional Switch Enabling the Recurrence of Malignant Phenotypes

AML is characterized by distinct cell populations; those cells with the potential for leukemia initiation were termed leukemia stem cells (LSCs), considered pivotal in the re-emergence of AML during relapse, endowed with an increased capacity for activation of NFκB, and associated with poorer clinical outcomes [76,77,78,79]. AML relapse is linked to the resistance of malignant cells to cell stress.

Specifically, cell stress contributes to feedback defects as it overrides modules that otherwise control NFκB activity to protect host tissue. As an example, oxidative stress augments NFκB activity, and leukemia cells can protect themselves either by maintaining high NFκB activity that is accompanied by expression of its downstream inflammatory target genes and metabolic genes, or by quiescence and suppression of the metabolism and by turning off sources of oxidative stress [80,81,82,83].

Uncovering the principles of NFκB function defects during recurrent neoplasia is expected to enable the refinement of the experimental design for agents aiming to neutralize key elements of resistant cancer. Recently, a screen of compounds for the inhibition of NFκB led to the selection of emetine as a potential anti-leukemia agent, with both in vitro and in vivo effects against human AML cells that were transplanted into NSG mice [84]. In NSG mice, similar effects were elicited with the proteasome inhibitor Bortezomib, with a clear impact on NFκB and a marked induction of oxidative stress, primarily affecting LSC [85]. Using piperlongumine as an inhibitor for NFκB showed that the cytotoxicity mainly affected LSC, without a comparable effect on normal bone marrow cells [86].

Cellular stress also causes phenotype changes and stemness-like gene expression in solid tumor cells. Either low folate or excessive supplementation with folate induces the expression of stemness-like genes in human adenocarcinoma cell lines [87,88]. Moreover, long-term treatment of hepatocellular carcinoma cells with the anti-neoplastic tyrosine kinase inhibitor Sorafenib induced the expression of *ALDH1A1*, *ABCB1A*, *CD133*, *Nanog*, *Oct4*, and alpha fetoprotein, and enhanced the capacity of cells to cause tumors as xenografts in mice [89]. Therefore, critical stemness properties of both leukemia and solid tumor cells are triggered after exposure to potentially cytotoxic conditions that cause cell stress.

## 5. Documented Impact of Chromatin Remodeling on Malignant Cells

In addition to the promoter and typical enhancer sequences, several genes associated with cellular phenotype (“identity”), are regulated by additional sequences, with lengths typically far over 10 kilobase pairs, and characterized by histone H3 acetylation at lysine residue 27; bound by the “histone-reader” BRD4, these enhancers may be induced by transcription factors, such as NFκB, and have a combinatorial effect on gene expression [90,91]. Having association with the cellular identity, these “super-enhancers” are highly relevant in cancer. A gene regulated by super-enhancer that has a particular relevance in leukemia, is *MYC* [92]. Its protein product MYC is considered an important target in the development of experimental treatments, because it permits rapid changes in cellular phenotype and metabolism [93,94]. However, research increasingly recognizes that perturbing chromatin accessibility is a key aim for experimental anti-leukemia drugs [94].

Histone-modifying enzymes have shown defects in malignant disease and are consequently targeted in developing experimental treatments [95,96,97]. Defects in the regulation of histone modifiers are also documented for NFκB target genes other than *MYC* [98,99,100,101,102,103]. Through interactions with chromatin-modifying enzymatic complexes, NFκB activates numerous genes involved in the mediation of inflammatory signals and in coordinating cell survival [104,105,106,107]. In particular, the bromodomain protein BRD4 has been implicated in NFκB transcriptional induction in response to diverse conditions that include oxidant stress. Consequently, BRD4 is targeted to inhibit NFκB transcriptional activity in a variety of cancer cells, and bromodomain inhibition has entered clinical trials [108,109,110,111,112] (NCT02543879).

Chromatin structure-associated tumor drivers include noncoding mutations at chromatin loop anchors and domain insulators, altered transcription factor binding, domain redistricting due to structural variation, and mutations in cohesin and metabolic genes. While metabolic products can alter chromatin structure–function relationships through enzymatic processes, there is evidence that non-enzymatic processes link metabolic outputs and chromatin structure as well [113].

Adenosine monophosphate-activated protein kinase (AMPK) is activated by metabolic stress and permits BRD4 placement to super-enhancers regulating leukemogenic genes; AMPK deletion reduced acetyl-CoA and histone acetylation, displacing bromodomain proteins from chromatin in leukemia-initiating cells. Furthermore, in both mouse and patient-derived xenograft AML models, treatment with AMPK and BET inhibitors synergistically suppressed AML [114]. Evolution of AML cells toward developing drug resistance, or toward relapse, entails convergence of their chromatin compaction status toward a compaction that is distinctive of LSC, which appeared highly conducive to increases in the expression of NFκB target genes and appears to be generally independent of the genetic state [115,116].

**Figure 2:**
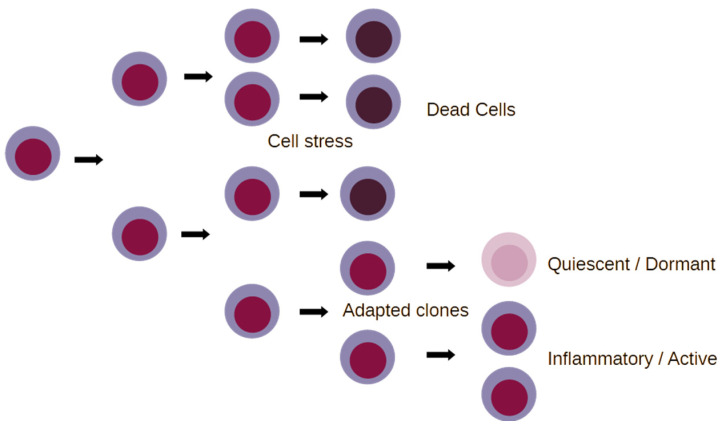
A simplified schematic of clone evolution for cancer cells under stress. Inflammation and cellular stress kill cancer cells by a number of different mechanisms. Defective responses to cell stress and inflammation, due to genetic or epigenetic inactivation of tumor suppressors under certain conditions, may permit the adaptation of cancer cells, which can give rise to either inflammatory or dormant cell clones. Specific aspects of chromatin accessibility, however, enable rather prompt switches between quiescent and inflammatory phenotypes in response to changes in tissue.

The way that the above information can be interpreted is that cellular stress adaptation of malignant cells, especially in the model of AML, converges chromatin status to an LSC-like state, which is conducive to the prompt activation of inflammatory gene expression, independently from the genetic assortment of the primary cancer (Figure 2).

**Figure 3:**
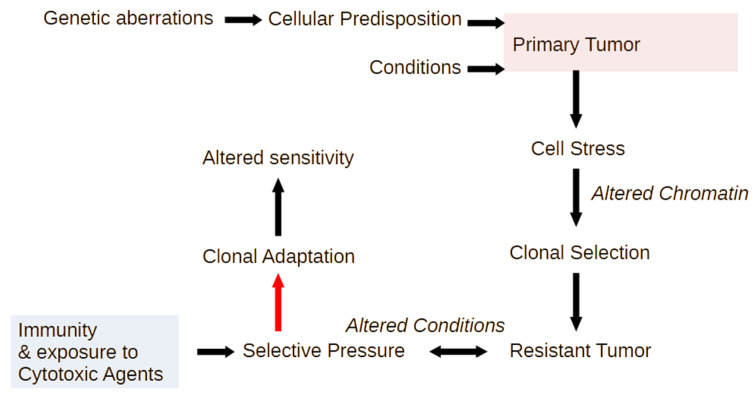
Inflammatory signal cascades generate conditions that restrict tumor growth, yet at the same time enable chromatin changes that permit phenotypic diversification and, consequently, the emergence of clones that are adapted to cell stress. Multiple different adapted clones, with diverse genetic assortments, may converge in the chromatin status, which is conducive to rapid changes in gene expression that permit adaptation to changing metabolic conditions and to altered interactions with the immune system. The type of malignant cell clones that are generated (red arrow) is critical to the severity of defects that these cells will cause to the host organism, ranging from paracrine effects to systemic defects in host immunity.

This means that cells during relapse have a chromatin state that enables substantial flexibility to respond to changes in the metabolic condition of host tissue, and this is clearly not limited to inflammatory genes, which are, however, a useful lead in the characterization of dynamic changes in cellular phenotypes. Therefore, the capacity of those cancer cells during relapse to react to challenging conditions is increased; this is mainly not due to a permanent genetic change (Figure 3).

Rather, the unfolded state of their chromatin permits rapid changes in gene expression, which deliver the assortment of proteins that permit those cells to respond to the given challenge. Increased expression of chromatin-modifying enzymes, and correspondingly increased transposase-accessible chromatin, are independent adverse prognostic factors for relapse in pediatric AML [117]. These AML cells are sensitive to proteasome inhibition and are mainly driven by NFκB activity and oxidant stress [85].

Chromatin accessibility also controls chemotherapy-induced dormancy and reactivation in solid tumors, with non-small cell lung cancer as an example, where cells surviving cisplatin chemotherapy entered dormancy [118]. The evolution of cancer cells under chemotherapy stress is regulated by transcription factors with binding sites initially buried deep within inaccessible chromatin. The transcription machinery and dynamic epigenetic alterations during the process of dormancy-reactivation of lung cancer cells after chemotherapy was investigated, using an assay for transposase-accessible chromatin sequencing (ATAC-seq). Global chromatin accessibility was extensively increased. Transcriptional Regulatory Relationships Unraveled by Sentence-based Text mining (TRRUST) v.2 was used to elucidate transcription factor–target interactions during the process of dormancy and reactivation. Enhancer regions and motifs specific to key transcription factors, including JUN, MYC, SMAD3, E2F1, SP1, CTCF, SMAD4, STAT3, NFKB1, and KLF4, were enriched in differential loci ATAC-seq peaks of dormant and reactivated cancer cells induced by chemotherapy.

Another line of evidence for the role of chromatin remodeling in paving the way for NFκB-driven cancer progression comes from glioblastoma, where NFκB selectively drives the expression of *EZH2* by activating its transcription; then, the final protein product, EZH2, once activated, causes a genome-wide change in methylated histone H3K27me3 expression and distribution. However, due to the pluripotent effect of canonical NFκB signaling, there was a synergistic cancer-promoting effect of the combination of NFκB and EZH2, which was detected both at the level of cell culture and cancer subcutaneous xenografts in mice, as well as at the level of glioblastoma patient prognosis, evident both in disease-free survival and in overall survival [102].

**Figure 4:**
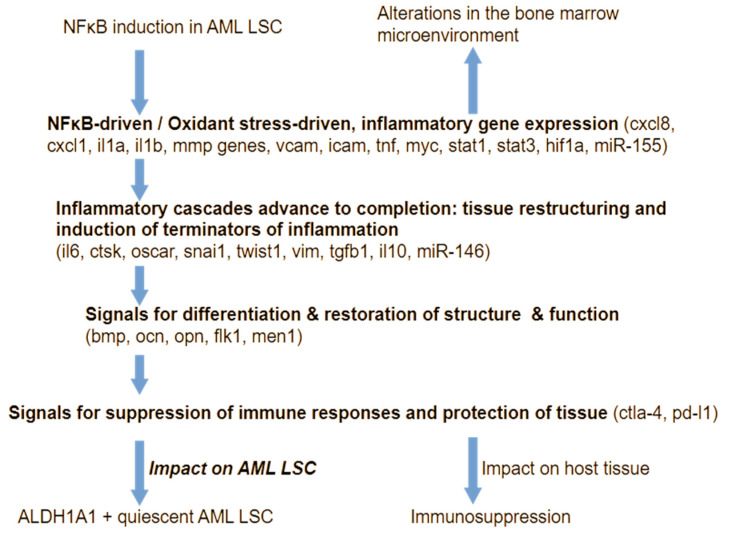
Gene expression is selectively unblocked in “stem-like” cells due to the accessible chromatin state, which permits the emergence of aggressive cancer (here AML) once a cell enters conditions that activate NFκB protein complexes. Cells that are not constitutively activating NFκB initially respond by positive feedback to inflammation, but ultimately activate negative feedback mechanisms that suppress inflammation and immunity, even if these cells are exposed to inflammatory stimuli. The nature of the cell signaling network architecture ensures redundancy in mechanisms for quenching inflammation. Cancer “stem-like” cells activate negative feedback to inflammation too, but incompletely, due to the perturbed functional state of their chromatin.

Thus, relapse in leukemia and solid tumors may be facilitated by increased chromatin exposure of cancer “stem-like” cells that survive cell stress by entering a state of dormancy/quiescence. The increased accessibility of chromatin allows for rapid changes in gene expression, which enable the emergence of new cell phenotypes that adapt better to the metabolic challenge. This phenomenon has a key consequence. Cancer cells upon induction of inflammation become decisively capable of transforming host tissue, and this affects the disease course (Figure 4).

Cells that do not have abnormal chromatin accessibility in regulators of inflammation respond according to the stimulus they receive, by inducing genes that amplify inflammation cascades and oxidant stress, and interfere with tissue function (such as inflammatory cytokines and adhesion molecules), and later gradually express new sets of transactivators, circulating mediators, and other molecules that set the stage for completion of inflammatory processes, to be followed by molecules that switch off the remaining mechanisms of inflammation and restore tissue function to finally express immune checkpoint molecules that protect host tissue from being damaged by the immune system [119,120,121,122,123,124,125,126,127,128,129,130,131,132].

During the last stages of this succession, immune tolerance is induced, and immune checkpoint molecules are expressed in a variety of cells, but, importantly, also in cancer cells [133,134,135]. At those stages, the expression of molecules that characterize quiescent cancer “stem-like” cells, such as the enzyme ALDH1A1, is also likely to accompany the expression of immune checkpoint molecules by malignant cells [136,137]. Importantly, however, due to their aberrant chromatin state, cancer “stem-like” cells can easily give rise to aggressive clones that revert to high expression of inflammatory genes, and that regain the capacity for rapid growth. Such rapid responses affect host tissue, impairing optimum function.

This allows cancer cells to escape destruction and to give rise to aggressive malignant clones, because their surrounding tissue cannot facilitate effective immune responses. As a result, the unidirectional generation of inflammatory mediators alters host tissue function. This is due to the fact that induction of inflammation causes in cancer “stem-like” cells practically unhindered expression of certain molecules, which alter tissue function by changing the phenotypes of surrounding cells drastically. It is important to note that the induction of inflammation in cancer “stem-like” cells also occurs as a result of the metabolic conditions that push intracellular signaling toward cell growth.

## 6. NFκB Is Involved in Pivotal Events during Cancer Development

NFκB, alone or in synergy with other transactivators, induces the expression of a variety of genes that in addition to inflammatory cytokines, encode modifiers of the extracellular matrix [138], and adhesion molecules [139,140], also include regulators of cellular protein turnover and cell death, as well as inducible transcription factors, such as hypoxia-induced factor 1 (HIF1α) [141,142,143], the proto-oncogene product MYC [144], Twist1 [145], and SNAI1 [146,147]. A substantial part of those gene targets themselves induce or repress NFκB activity depending on the conditions and the cell phenotype. This mutual regulation enables the generation of feedback loops that either enhance or suppress inflammation.

HIF1α was shown to mediate stemness in cancer cells [148], so what would be the role of NFκB in general, if it is upstream of HIF1?

In human induced pluripotent stem cells, treatment with p65 siRNA abolished the expression of the undifferentiated markers Oct3/4 and Nanog and upregulated those of the differentiation markers WT-1 and Pax-2 [149]. Thus, NFκB is also clearly involved in promoting stem-like properties, in spite of its core role in inflammation. This happens because, after inflammation, the stem cell pool needs to be replenished. This would occur in normal tissue; however, in cancer tissue it could be perturbed to enable the plasticity of cancer “stem-like” cells. If we add a mutual capacity for interference between NFκB and steroid hormone receptors to this effect, and amplification of inflammatory and growth signals by transactivator AP-1, it becomes clear that drastic effects on cell differentiation status and on host tissue function ensue upon the loss of critical restrictions in p65 activity [150].

Perturbation of the inflammation vs. regeneration functions of p65 might be illustrated in the interactions with STAT proteins, which are encoded by p65 target genes: the NFκB target gene products STAT1 and STAT3 regulate inflammation, and STAT3 may decrease interactions between IκBα and RelA and thereby provide critical feedback in NFκB-driven gene expression, affecting the subsets of the NFκB target genes that are expressed [151,152,153,154,155,156]. Events, like this STAT3-p65 interaction, have profound effects on gene expression.

Additionally, in cancer cells, specific alterations in their chromatin state may cause certain genes to be aberrantly activated by NFκB without the Rel-subunit phosphorylation that is normally needed to recruit histone-remodeling complexes [27]. This means that critical genes that normally require multiple steps to be expressed are now aberrantly induced and cause the increased resistance of cancer cells to cell death that would normally be triggered by the immune system or by pharmaceutical substances. This happens because these cancer cells, due to their perturbed chromatin status, adopt partial gene expression programs for tissue regeneration. They express genes that are intended to protect host tissue, which turn to protect at least part of the neoplastic tumor. Furthermore, the deregulated expression of inflammatory mediators can be expected to increase oxidative stress and, thus, DNA damage in host tissue, in addition to oxidative damage in a variety of macromolecules, which triggers a number of rescue mechanisms for the replenishment of damaged biomolecules and organelles [41,83]. These mechanisms are exploited by cancer cells to resist cell death.

A comprehensive list of genes (updated until 2010) regulated by NFκB is provided by the Gilmore Lab at https://www.bu.edu/nf-kb/generesources/target-genes/ (accessed on 6 July 2024).

All of these facts provide evidence for NFκB’s involvement both in suppressing cancer through the immune system and in promoting cancer by neutralizing the immune response and by blocking a variety of cytotoxic pathways in cancer cells. The way to reconcile all of the above is to emphasize that NFκB complexes provide an inducible platform for the relay of signals that initially promote inflammation and finally terminate inflammation to protect host tissue [157]. This is also evident in the involvement of NFκB in the changes between macrophage phenotypes during inflammation and cancer, leading to the proposal to reshape experimentally and ultimately therapeutically the tumor microenvironment via terminating macrophage recruitment [158].

Cytokines, such as tumor necrosis factor (TNF), mediate decisive interactions between the cancer and stroma, with characteristic experiments performed at the lab of Ben-Baruch demonstrating that TNF-induced NFκB p65 RelA and TGFβ1-induced SMAD3 acted in parallel in mesenchymal cells to activate the expression and release of factors that affect other cells in their microenvironment and induce breast tumor cell elongation, migration, and scattering out of spheroid tumor masses [159,160]. In breast cancer and in prostate cancer, NFκB-driven signaling activates positive feedback signals between cancer and stromal cells, whereby canonical, p65-driven signals form positive feedback loops with the non-canonical NFκB pathway, increasing resistance to hormones and to endocrine therapy [161]. In cancer cells, p65-driven gene expression conferred metabolic plasticity [162]. Oncogene *RAS*-induced cell transformation and the acceleration of aerobic glycolysis in p53-deficient cells required p65 expression [163].

It is very important to note that a number of key interactions of NFκB with proto-oncogene products occur at the level of gene expression when both NFκB and tissue regeneration drivers, such as TGFβ1 and MUC1, are simultaneously active [27]. NFκB, TGFβ1, and MUC1 interact to change the cellular phenotype, lift growth restrictions, and deregulate angiogenesis and immunosuppression [51]. Even though a short interval of coexpression is part of the physiological transition between inflammation and the return to tissue normal function (re-establishment of homeostasis), in those cancer cells that have lowered restrictions on NFκB activity, the result is that the coexpression of inflammation mediators and tissue regeneration and dedifferentiation genes causes a substantial transformation of the host tissue. For example, TGFβ1 has a key role in phenotypic transitions of leukemia cells that affect their microenvironment [164].

NFκB also activates the expression of the micro RNA species miR-155, a species overexpressed in many types of cancer, which drives aneuploidy at early stages of cellular transformation [165,166]. RelA p65 is essential for miR-155 induction in hepatocellular carcinoma [167]. Micro RNA are short non-coding RNAs thatmediate the repression of other genes by interference with them at the RNA level based on sequence complementarity. One micro RNA species may bind to the 3′ untranslated (3′UTR) regions of several target messenger RNA, and thereby inhibit the gene expression of multiple gene targets within the same or different signal transduction pathways. Therefore, the impact of p65 in micro RNA species amplifies the extent of the network of genes and cellular functions affected by NFκB. However, the micro RNA miR-155 and miR-146 provide two different modes of feedback regulation to NFκB [168].

The combined action of “mostly positive” (miR-155) and “mostly negative” (miR-146) NFκB-miRNA feedback loops fine tunes the NFκB activity during inflammatory processes, and eventually leads to the resolution of the inflammatory response [169]. It must be noted that “mostly” is here used to emphasize that micro RNA expression and their gene targets are highly phenotype-specific and, therefore, can never be expected to remain inert, as a given micro RNA species may act against inflammation in most cell types, and have the opposite effect in other cell types, depending on the assortment of signal transducers that operate in a cell at a given timepoint. We focus here on miR-155 and miR-146, although there are a substantial number of micro RNA species that interact with NFκB (reviewed in [169]).

**Figure 5:**
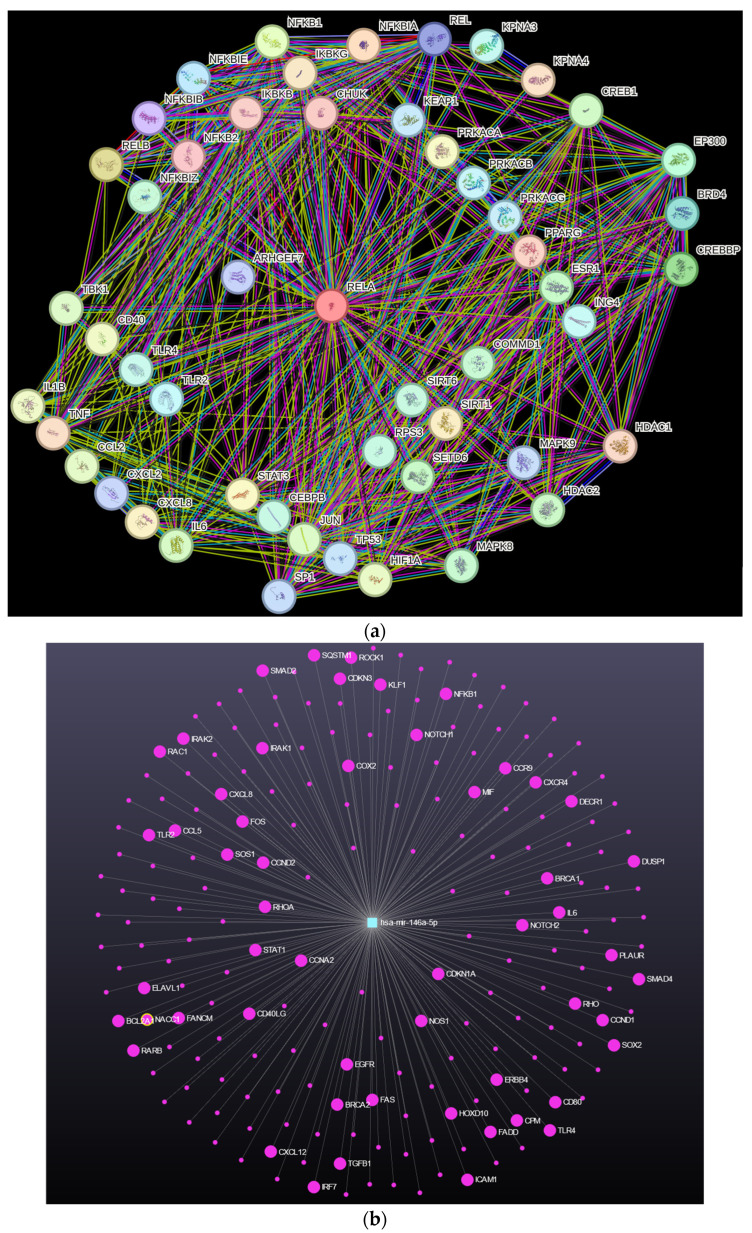
Illustrating the complexity of feedback mechanisms helps understand the depth of the impact of inflammation in cell signaling, which affects both conditions within the cell, as well as between the cell and its surrounding tissue. (**a**) A selection of “high-confidence” interacting proteins/genes with NFκB p65 (RelA) obtained with the platform string (https://string-db.org/, accessed on 6 July 2024). Details are clarified in the Supplementry Materials Figure S1. (**b**) An example of a key NFκB feedback gene is the micro RNA species *miR146*. This gene is prospectively associated with numerous other genes that influence cell stress responses (such as *SQSTM1*), inflammation (such as *CXCL8*), and cell phenotype (such as *TGFβ1*). Source miRNet 2.0 (https://www.mirnet.ca/, accessed on 6 July 2024).

In general, abnormal NFκB control allows cancer cells to exhibit high levels of transcriptional and phenotypic plasticity [170,171], by altering their chromatin state and thereby permitting adaptation to cell stress and curtailing the dependence of cell survival on feedback signals from the host tissue [27,172]. This means that cancer cells can adapt their gene expression profiles and phenotypes in response to changing environmental conditions, aiding in their survival, increasing the phenotypic diversity of cancer cell subclones, and licensing proliferation on metabolically adapted cancer cell subpopulations (a selection of p65 interactants is given in Figure 5a). Importantly, aberrant feedback to NFκB from its downstream target genes increases the functional dichotomy between tumor and non-tumor cells in response to cell stress. The downstream target gene *miR146*, for example, influences a wide spectrum of other genes that mediate the effects of cell stress and inflammation (Figure 5b) [173,174].

miR-146 is experimentally tested as an inhibitor of NFκB-mediated effects in the development of cardiac and bone pathology [175,176]. In breast cancer cells, miR-146 acts as a negative feedback effector for NFκB, yet the downstream effects of miR-146, and its potential as a tumor suppressor, are cell-dependent [177].

In contrast, in both normal and breast cancer cells, miR-146 supports “stemness”, with target genes in pathways and cellular functions for the exit from quiescence (activation of the oxidative phosphorylation metabolism, of the G2–M transition, E2F targets, and cell cycle) and to transcriptional programs that control the stem cell phenotype (inflammatory pathways, hypoxia, and epithelial-to-mesenchymal transition) [178]. miR-146 is an endogenous inhibitor of inflammation and myeloid cell proliferation; however, its potential as a tumor suppressor evidently depends on the metabolic state of cells [179].

This means that cancer cells can survive and thrive during nutrient deprivation, hypoxia, and immune responses by leveraging aberrant feedback to inflammatory stimuli and cell stress, and possibly bypassing physiological inhibitors of oncogenesis, such as miR-146. The process that couples cell stress to adaptation can physiologically entail sequential activation and inhibition of NFκB, and ultimately lead from metabolic stress to the induction of antioxidant defense [180,181,182]. However, the documented function of NFκB adjustment in physiological adaptive processes underscores its pivotal involvement in cancer development, progression, and resistance to therapy, when feedback or general restriction to NFκB is impaired [111,114,183,184]. The evidence of the effects of p65 signal networks in metabolic adjustment of malignant cells highlights the potential of characterizing and experimentally targeting NFκB pathways to disrupt the adaptive mechanisms of cancer cells. This approach should ultimately deliver better options to improve therapeutic outcomes.

## 7. The Impact of NFκB-Associated Chromatin Remodeling on Immunity

AML stem-like cells that are most likely to cause relapse can readily activate NFκB-driven gene expression due to their remodeled chromatin. However, this has an impact on the rest of the organism, and more than anything else, on their microenvironment. AML cell mutations already prime leukemia cells for inhibition of the immune response. For example, mutated isocitrate dehydrogenase causes the accumulation of D-2-hydroxyglutarate in leukemia cells, which triggers HIF-1a protein destabilization, resulting in metabolic skewing towards oxidative phosphorylation, increased regulatory Tcell (Treg) frequency, and reduced T helper 17 (Th17) polarization [185]. D-2-hydroxyglutarate also inhibits IL-12 secretion by dendritic cells [186] and suppresses antitumor T cell immunity by (a) inhibiting expression of antigen receptor HLA-DP in AML cells, and (b) impairing human dendritic cell differentiation, resulting in a tolerogenic phenotype with low major histocompatibility class II expression [187]. However, 2-hydroxyglutarate accumulation can also cause aberrations in the regulation of chromatin remodeling and oxidant stress, which could also predispose mutated isocitrate dehydrogenase AML cells to a divergent response to inflammation, with a selective NFκB-driven impact on stromal cells [188,189].

While AML cells may activate NFκB beyond the normal limits that are dictated by tissue function, their constitutive secretion of inflammatory mediators can lead to the inhibition of NFκB in immune cells, due to physiological negative feedback, impairing T-cell effector function [190]. This has negative effects on the disease course. In favorable AML, the leukemia cells are largely dependent on mediators secreted by cytotoxic CD8 T cells for signals activating stemness, cell proliferation and cell survival, immune-related signaling, and growth factor signaling. However, in unfavorable AML, leukemia cells develop autocrine signaling pathways and grow independently from CD8 T cells [190], which apparently paves the way for disease worsening.

AML cells in bone marrow also stimulate mesenchymal cells to cause an increase in the frequency and activity of immunosuppressive regulatory T cells [191]. However, the interaction with regulatory T cells is not exclusively allowed only to AML cells. In fact, normal aged hematopoietic stem cells that accumulate genetic mutations entrap regulatory T cells to shape their microenvironment toward conditions favorable for their own survival [192].

Similar to AML interaction with T cells, leukemia cells also suppress NFκB-driven inflammatory gene expression in macrophages to polarize them toward the immunosuppressive M2 phenotype. This macrophage polarization can be reversed by inhibiting efferocytosis, the immunosuppressive process whereby macrophages phagocytose apoptotic cells [193].

In general, during the early stages of cancer development, M1-like anti-tumor macrophages infiltrate tumors. This is followed by their subsequent polarization into pro-tumor M2 macrophages later in the course of the disease, along with myeloid-derived suppressor cells [194]. This M1 to M2 switch occurs through sustained exposure to polarizing factors released by the cancer cells and direct cell-to-cell contact between cancer cells and macrophages [195]. However, asymmetry in signal processing between cancer cells and stromal cells can also result in damage to host tissue, which is especially evident in advanced adenocarcinoma [109].

In conclusion, the remodeled chromatin of advanced cancer cells functions in a manner that enables swift changes in key phenotypic aspects that enable the protection of at least part of the malignant tumor from the immune system, by altering interactions of the tumor with almost every type of immune cell, and on many different levels. A general assessment of tumor-infiltrating immune cells shows that cancer progression depends on whether NFκB is activated in immune cells or in cancer cells [196]. This necessitates developing effective methodologies to track the impact of tumors on the immune system. These methodologies need to be rigorously tested with the aim of ultimately reaching routine use.

## 8. Preclinical Effects of Inhibiting Bromodomain Proteins and Their Downstream Targets in AML

Inhibiting the activity of bromodomain proteins by directly causing their degradation in AML cells killed both AML cell lines as well as primary AML blast cells directly isolated from patients. In those experiments, bromodomain and extra-terminal protein degradation was induced by ARV-825, the heterobifunctional small-molecule degrader of BET proteins, which contains a ligand for a BET protein connected via a linker to a ligand for the E3 ubiquitin ligase cereblon. AML cell killing was manifest in the submicromolar range and was accompanied by a series of biological effects [197]. At least a portion of these events were predictable, especially the decrease in MYC expression, as well as a decrease in the expression of survival proteins (B cell leukemia/lymphoma 2 [BCL-2], myeloid cell leukemia sequence 1 [MCL-1], etc.) and the decrease in the expression of the MYC downstream target PIM1. ARV-825 not only downregulated pro-survival proteins it also suppressed surface expression of CXCR4 (not total CXCR4) and CD44 in the LSC compartment. Additionally, ARV-825 reduced intracellular cystine, increased cellular oxidant stress (ROS), and downregulated the expression of genes associated with the LSC signature and the Wnt/β-catenin pathway. Consequently, ARV-825 reduced the LSC burden and improved survival in a mouse model of disseminated AML that was studied both with luciferase-transduced AML cells, as well as with patient-derived xenografts. It is important to note that ARV-825 gave better anti-leukemic activity in combination with cytarabine than when used alone.

Coculturing of AML cells with healthy donor bone marrow-derived mesenchymal stromal cells (NMSCs) and treating them with ARV-825 or cytarabine under normoxic or hypoxic conditions rendered AML cells relatively resistant to cytarabine. Conversely, sensitivity to ARV-825 was the same in mono- or cocultures and under both O_2_ conditions; thus, ARV-825 overcame both stroma- and hypoxia-mediated resistance at least to some extent.

At the RNA level, ARV-825 caused a substantial change in the gene expression of both primary as well as cultured AML cells, with over 1000 downregulated genes and over 700 upregulated genes meeting the significance criteria (*p* ≤ 0.01) for a ≥1 change in the log2 value. It is important to note that compared with AML cells, ARV-825 exposure of NMSCs induced a relatively smaller change in the transcriptome: 340 downregulated genes and 140 upregulated genes met the significance criteria.

**Figure 6:**
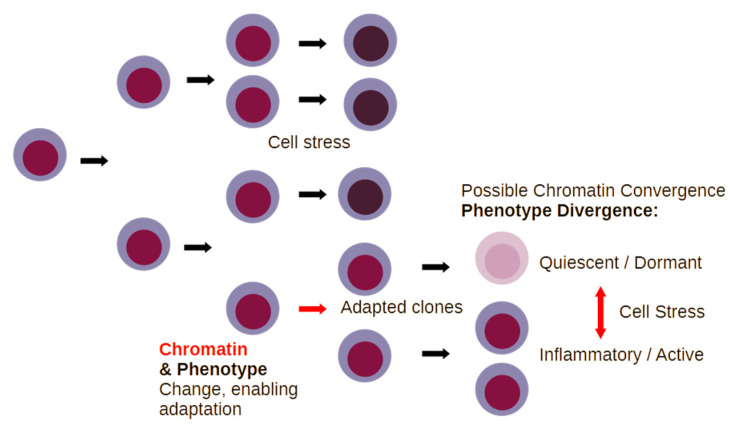
A divergent response to cell stress characterizes malignant cells and shapes the pool of surviving malignant clones after changes in the conditions of their microenvironment. This phenomenon is especially evident in recurrent cancer. An additional reason why this is important lies in the fact that by proliferating, cancer cells gain the capacity to generate more phenotypically or genetically diversified clones (red arrows). By increasing clonal diversity, cancer becomes difficult to eradicate.

All of the above reinforces the notion that at the level of chromatin-reader BET proteins, cellular stress, as modeled here by cytarabine treatment and ROS, causes divergent responses in malignant cells when compared to non-malignant cells (here NMSCs), and clearly affects the interactions between them. A divergent response to cell stress marks, therefore, a biological property of malignant cells and is tractable at least at the level of chromatin reader BET proteins (Figure 6).

## 9. Evidence against a Unidirectional Effect of Cell Stress and NFκB Signaling in Cancer

Estrogen-deprived breast cancer cells when exposed to estrogen undergo apoptosis that is driven by NFκB, which is inhibited by glucocorticoids [198]. Treatment with antiestrogens initially induces mild UPR through estrogen receptor ERα with the activation of three sensors of UPR-PRK-like endoplasmic reticulum kinase (PERK), inositol-requiring enzyme 1α (IRE1α), and activating transcription factor 6 (ATF6) in the endoplasmic reticulum. Subsequently, these sensors interact with the transcription factors MYC, NFκB, and HIF1α, leading to acquired endocrine resistance. Paradoxically, estradiol (estrogen E2) further activates sustained secondary UPR via ERα to induce apoptosis in endocrine-resistant breast cancer. Specifically, persistent activation of PERK triggers the NFκB/TNF axis, ultimately determining cell fate to apoptosis [199]. Inflammatory cytokine gene expression gave a positive prognostic effect associated with the activation of innate and acquired immunity [200]. Furthermore, p65 inhibition led to cancer stem cell enrichment in hormone receptor-positive/HER2-negative breast cancer cells, and this effect was mimicked by a STAT3 mutant deficient in the activation of p65 transcriptional activity [201].

Therefore, the assortment of signaling modules in cancer and stromal cells determines the effects of cell stress and inflammatory gene expression. Especially in regard to cancer stem-like cells, inflammation has the natural effect of restraining their growth and activating immune responses; however, this depends on the degree of cancer stem cell adaptation and the immune modulators expressed. Evidently, in relapse and in cancer refractory to treatment, these natural mechanisms are overridden by modules that become available by chromatin unfolding. The degree to which cell stress and NFκB can restrain tumor growth depends on the degree to which their networks are connected to tissue homeostasis [27].

This evidence challenges the notion of a unidirectional effect of cell stress and NFκB signaling in cancer. The interaction between cell stress, NFκB signaling, and cancer progression is complex and context-dependent, with the potential for both tumor-suppressing and tumor-promoting outcomes. This underscores the importance of considering the specific cellular and molecular context when targeting these pathways for cancer therapy.

## 10. Prospects for Marking NFκB and Developing Intervention Methods

NFκB activity is not triggered by a single inducer. NFκB signaling is still detected in samples from cancer patients that do not respond to treatment, even if this treatment involves last-generation agents, such as the tyrosine kinase inhibitor afatinib [202]. In fact, it was proposed to focus on NFκB in order to overcome the problem of tumor signaling pathway heterogeneity in osteosarcoma [203]. This can be achieved if enough good data enable a rigorous assessment. As we discuss below, there are a number of options available, and the progress that we can expect from novel technologies and improved parameter detection and registration should lead to even more effective intervention options.

The obvious question that arises concerns where best to focus when the exposed chromatin allows cancer cells unlimited options in phenotype changes. The answer is probably to exploit the inherent defects in cancer cell metabolism, and specifically the enzymes and organelles that are defective due to the very nature of cancer. Cancer cells develop specific adaptations in lysosomes, mitochondria, or other structures, such as stress granules [204] due to the products of the defective neoplastic genome. These adaptations lead cells to enter a state of death-predisposed metabolic function that can be experimentally converted to cell death. For example, by using agents that interfere with lysosome function or with mitochondrial function, it is possible to kill resistant breast cancer stem cells [205,206]. Killing cancer stem cells is also possible by interfering with activators of NFκB, such as the enzyme ataxia telangiectasia mutated (ATM) kinase that sets in motion multiple mechanisms fine tuning organelle function to generate cancer stem cells [207]. Another key target group are proteins, such as SRC3, that mediate signal interference between steroid receptors and NFκB, and which give promising experimental results [208].

Still, there is the problem of remodeled, dysregulated chromatin in advanced cancer that needs to be addressed. To this end, a number of experimental and clinical interventions aim to disrupt the chromatin remodeling of cancer cells. BET inhibitors continue to be developed and tested with the aim of disrupting NFκB-dependent gene expression [209,210]. In addition to BET inhibitors, histone deacetylase inhibitors, such as RGFP109, block NFκB-dependent transcription in cancer cell lines and thereby overcome resistance to chemotherapy [211]. Furthermore, micro RNA species, such as miR146, are experimentally targeted to inhibit NFκB-dependent inflammation in myeloid cells and leukemia progression in malignant cells [212]; it must be noted here that micro RNA effects are highly cell-dependent and, therefore, caution must be applied in using them as NFκB inhibitors.

A wide range of natural substances have been experimentally tested for the inhibition of NFκB activity and may proceed to the clinic once key aspects of their pharmacodynamic behavior are successfully addressed, with the most notable example being curcumin, which may become a powerful anti-inflammatory agent [213,214,215,216,217,218,219]. Furthermore, probiotics decrease NFκB activity and appear to favor the survival of cancer patients [220]. Another strategy to inhibit cancer-promoting effects of inflammation is to block the recruitment of macrophages, since this cell type is involved in a great part of the suppression of the immune response [158].

In general, a great number of diverse substances have been tested and shown to inhibit NFκB with a potential therapeutic relevance in leukemia; these include upstream-acting substances, such as proteasome inhibitors, IKK inhibitors, inhibitors of serine/threonine kinases, such as GSK-3β [221], further upstream tyrosine kinase inhibitors, as well as flavonoids and sesquiterpene lactones that inhibit either IKK or p65 or both, inhibitors of the p65 interactome, and also inhibitors of downstream targets, such as the BCL2 family antiapoptotic proteins [79,222]. However, this field is rapidly evolving, with an increasing number of interacting modules targeted by experimental agents [223].

Since a great number of NFκB effects occur in the microenvironment through the secretion of cytokines, adhesion molecules, and matrix-modifying enzymes, it is plausible that a variety of methodologies need to be used to study aspects of inflammation signaling in the microenvironment, such as three-dimensional model systems that examine the interaction between different cell types in microenvironments that mimic host tissue [224]. A notable example is the development of bone marrow organoids for research into the impact of leukemia on the bone marrow, which may permit precise targeting of the mechanisms underlying leukemia resistance to therapeutic intervention and to the immune system [225]. The wider application and improvement of single-cell analysis both in clinical samples and in advanced model systems allows for determining the proportion and response dynamics of cancer-initiating cells, which immediately suggests the appropriate methodology for their destruction [226].

## 11. Use of NFκB as a Target or Biomarker in Cancer Clinical Trials

NFκB is the target in a number of studies of cancer treatment. Over a decade ago, it had already become evident that inhibition of the proteasome alone could not inhibit NFκB in AML [77]. High proteasome activity, which positively regulates NFκB activity, is often observed in AML patients [227], but inhibiting the proteasome can activate cell stress responses in cancer cells and enable alternative pathways for proteolysis of IκBα, such as the lysosome [41]. A number of different trials have begun in recent years, aiming to evaluate NFκB as a biomarker in cancer, and to assess a variety of conditions and treatments for NFκB inhibition.

The randomized phase II trial NCT02144675 will evaluate how well choline magnesium trisalicylate with idarubicin and cytarabine works in treating patients with AML. The study will determine if salicylate alters the expression of NFκB-regulated genes in AML cells and if NFκB modulation by salicylate alters AML chemotherapy drug efflux.

Study NCT03978624 aimed to determine changes in NFκB and STAT3 in muscle-invasive bladder cancer after the use of the humanized antibody pembrolizumab and the selective class I histone deacetylase (HDAC) inhibitor entinostat. The results are awaited.

NCT00503841 aimed to estimate the effect of the EGF receptor inhibitor erlotinib on the expression of nuclear NFκB and amphiregulin in patients with ER-negative, EGFR-positive, and IL-1α-positive breast cancer. EGF receptor was expected to drive constitutive activation of NFκB in adenocarcinoma cells [228]. However, NFκB activation by other stimuli may mediate resistance of cancer cells to EGFR inhibitors [229].

Study NCT02546440 was aimed at assaying the NFκB inhibiting and apoptosis inducing drug Dimethylfumarate (DMF) in patients with cutaneous T cell lymphoma. NFκB inhibition was achieved, and the group will proceed toward a phase 3 trial [230].

BMX-001, a redox active metalloporphyrin is designed to mimic the center of superoxide dismutase. The primary mechanism of action is the modulation of cellular signaling pathways. BMX-001 inhibits both NFκB and HIF-1α. By inhibiting these pro-survival and pro-angiogenic transcription factors, BMX-001 augments tumor killing by radiation therapy and inhibits tumor regrowth. The inhibition of NFκB blocks major components of the inflammatory cascade, which simultaneously results in the protection of normal tissue from radiation-induced injury. BMX-001 is also being developed in head and neck cancer, anal cancer, and rectal cancer, and has been previously granted Orphan, Fast Track, and Breakthrough designations by the FDA [231,232,233,234].

A Phase 1 trial of CA-4948, a small-molecule IRAK4 kinase inhibitor, and ibrutinib, a Bruton’s tyrosine kinase inhibitor combination in patients with relapsed or refractory hematologic malignancies has begun. Ibrutinib targets one of the two main pathways activating NFκB in Bcell malignancies.

Biomarker correlations, such as MYD88-L265P mutations, IRAK4 pathway, and NFκB inhibition, will form exploratory objectives [235]. The inhibitor of nuclear export Selinexor in combination with ibrutinib was a tolerable treatment for patients with chronic lymphocytic leukemia and non-Hodgkin lymphoma in a phase 1 study [236].

For follicular lymphoma, an intravenous inhibitor for phosphatidylinositol-3-kinase is approved from the FDA for the treatment of relapsed patients who had received at least two prior systemic therapies [237]. In lymphoma, this inhibitor is aimed at blocking cancer cell NFκB activation.

NFκB activity is the primary outcome measure that will be used to evaluate treatment of aspirin as well as metformin to prevent recurrent colorectal cancer after surgery (NCT03047837).

In a study of the proteasome inhibitor bortezomib and the antiviral raltegravir for human T cell leukemia virus-associated adult T cell leukemia lymphoma, in patients that achieved a partial response to therapy, the most affected NFκB targets were more likely to be repressed after therapy than in patients with progressive disease [238]. This points to NFκB as the underlying mediator of severe disease course, at least during the time course of monitoring.

Antioxidant N-acetylcysteine was intended to inhibit NFκB in the tumor microenvironment in patients with persistent or recurrent high-grade ovarian, primary peritoneal, or fallopian tube cancer in clinical trial NCT02569957, but the study was halted prematurely due to slow accrual.

NFκB will be assayed as a tumor activation marker in a trial of the combination of chemotherapy and an anti-hypertensive, aiming to suppress myeloid-derived suppressor cells (MDSCs) in patients with resectable gastric or gastroesophageal junction adenocarcinoma (NCT05709574).

Proteasome inhibitor bortezomib was beneficial in de novo pediatric AML patients with low phosphorylation of NFκB [239]. Activating the phosphorylation of NFκB is a pathway for proteasome-independent NFκB activation [240,241]. This demonstrates the clear need for effective NFκB inhibition in patients with high constitutive phosphorylation of the NFκB subunit RelA, which are resistant to proteasome inhibitors. It is very important that RelA phosphorylation was convincingly shown to determine the effect of bortezomib, since inhibiting proteasome can also have NFκB-independent effects on cell survival, such as stabilizing the apoptotic protein BIM [242,243].

On the contrary, NFκB was not assayed in a trial of bortezomib combined with homoharringtonine and cytarabine for refractory or relapsed AML, which determined the treatment as tolerable [244]. As such, the aspect of the molecular response remains to be determined.

## 12. Plasticity of Cancer “Stem-like” Cells May Have a Clinical Impact in AML: ALDH1A1 Is outside of the Box

The clinical impact of phenotype plasticity of cancer stem-like cells becomes visible when examining *ALDH1A1* gene expression in different risk groups of AML, and when comparing primary and recurrent disease. Plasticity is a characterized property of AML stem-like cells, and the emergence of clones with high *MYC* expression is rather common [245,246,247]. NFκB and BRD4 are recognized as a potent drivers of *MYC* expression in AML stem-like cells [245,246,247,248]. Among other effects, MYC increases ribosomal biogenesis to enable the capacity of AML cells to proliferate [249]. AML stem-like cells that are most likely to cause relapse may readily activate NFκB-driven gene expression due to their remodeled chromatin, exclusively overcome G1 arrest of the cell cycle, and express antiapoptotic factors, such as BCL2 [250,251,252].

What is interesting is that the overexpression of RNA from the gene *ALDH1A1* that encodes an aldehyde dehydrogenase that is well suited to protect quiescent stem cells from oxidant stress has a negative prognosis in AML, despite the fact that this gene would be expected to mark leukemia stem cells that do not operate MYC-driven metabolic circuits [82,253,254]. The way to reconcile this observation is to envision a dynamic continuum of phenotypes of leukemia stem cells, whereby the few cells that survive chemotherapeutic intervention may be quiescent; but upon favorable conditions and an inflammatory stimulus, due to their exposed chromatin, they instantly induce *MYC* gene expression. Making MYC instantly available means that they can readily express MYC-driven metabolic genes and proliferate, giving rise to overt disease [83]. Thus, a steady-state high level of RNA from the *ALDH1A1* gene increases the capacity of leukemia cells to respond adequately to adverse conditions but does not prevent leukemia “stem-like” cells from generating subclones with high *MYC* expression and high metabolism that can give rise to aggressive disease.

Inhibiting ALDH1 selectively kills leukemia stem cells without killing normal hematopoietic stem cells, which is a hint of a continuum of phenotypes that operates exclusively in malignant “stem-like” cells [255]. It will be interesting to learn if ALDH1A1 protein expression, by increasing the available activity of this ALDH enzyme, actually turns out to be a “last-minute” shield for death-predisposed AML cells. If this is true, then it opens the way for learning critical details of the basic pathways of selection of resistant cancer cells and thereby delivers an advanced picture of the biological processes leading to cancer relapse.

## 13. Conclusions: The Dynamic Impact of Cell Stress on Cancer Stem-like Cells Permits Relapse

The substantial impact of BET protein-targeted experimental interventions is promising. However, it is crucial to recognize that malignant cells can evade treatment by entering a quiescent, dormant state with corresponding metabolic adaptations. When tissue conditions in the tumor microenvironment become permissive for growth once again, they favor cancer cell growth, and these adapted malignant clones may divide to generate a diverse array of subclones, which can cause the recurrence of neoplastic disease. This dynamic evolution of cancer stem-like cells is facilitated by their chromatin state, which permits rapid changes in gene expression. Using the term “stem-like” allows us to cover variations in the genetic assortment, which may converge into a number of essential properties. Thus, it is important to consider the fact that malignant cells, through diverse stimuli and sources of stress, may enter quiescence, a dormant state with corresponding metabolic adaptation.

At the heart of this process lies NFκB, a redox-sensitive transcription factor activated by cellular stress. NFκB orchestrates a multifaceted program, driving the transcription of genes that promote cell survival, inflammation, and resistance to cell death. This creates a self-reinforcing loop, where inflammatory signals establish autocrine and paracrine pathways that further enhance malignant cell survival and proliferation. The flexibility and adaptability of cancer stem-like cells to cell stress, mediated by NFκB and other factors, underscore their role in cancer relapse.

In essence, cancer stem-like cells exploit cell stress not inevitably as a dead end, but as an opportunity to shape their environment and enhance their fitness for relapse. Understanding the dynamic impact of cell stress on cancer stem-like cells and the mechanisms underlying their chromatin state and signaling pathways is essential. This knowledge can inform the development of more effective experimental methods to prevent cancer recurrence by targeting the specific pathways that allow these cells to survive and thrive under adverse conditions. By unraveling the intricate interaction between cell stress, chromatin remodeling, and NFκB signaling, we can ultimately develop more effective therapeutic strategies that not only target the initial tumor burden but also eradicate these elusive cancer stem-like cells, finally achieving durable remissions.

## Supplementary Materials

The following supporting information can be downloaded at: https://www.mdpi.com/article/10.3390/ijms25168621/s1.
