# Supplementary figures and images for: Divergent Processing of Cell Stress Signals as the Basis of Cancer Progression: Licensing NFκB on Chromatin
Source: Int J Mol Sci. 2024 Aug 7;25(16):8621. doi: 10.3390/ijms25168621 (PMC11354898; doi:10.3390/ijms25168621)

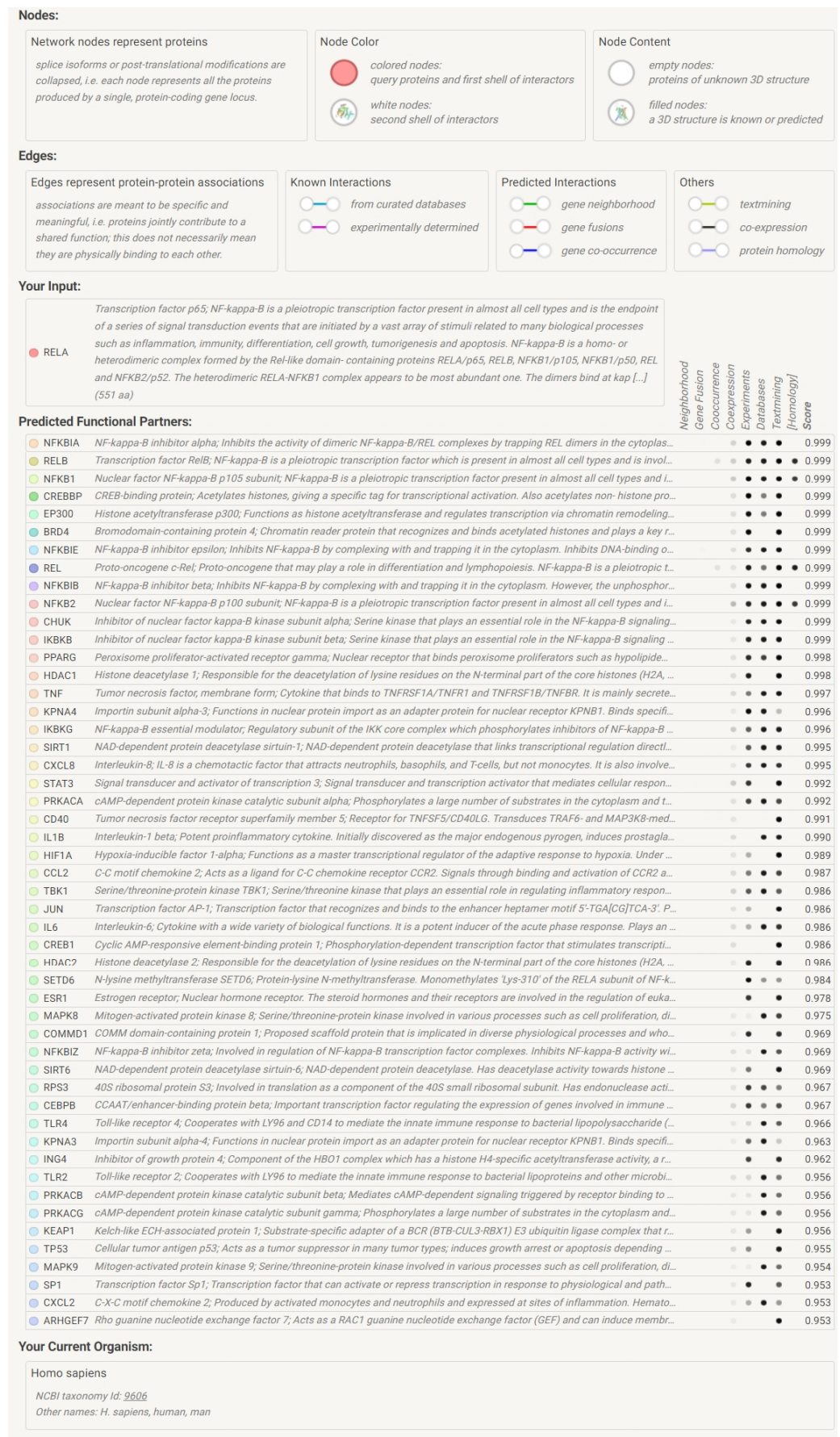

**Figure S1.** Detailed description of the content of Figure 5a.

Supplement: Supplementary file 1 [file ijms-25-08621-s001.zip › ijms-3124210-supplementary.pdf]
